# Analysis of Gut Microbial Communities and Resistance Genes in Pigs and Chickens in Central China

**DOI:** 10.3390/ani12233404

**Published:** 2022-12-02

**Authors:** Yapei Rui, Gang Qiu

**Affiliations:** College of Animal Science and Veterinary Medicine, Xinyang Agriculture and Forestry University, Xinyang 464000, China

**Keywords:** gut microbial communities, resistance genes, pig, chicken, China

## Abstract

**Simple Summary:**

Objectives: The goal of this study was to reveal the gut microbiota of pigs and chickens in central China and the dynamics of antibiotic resistance genes carried by microorganisms. Methods: Free range and factory-farmed Gushi chickens and Huainan pigs were divided into eight groups. Faecal samples were collected from each group, and the metagenomic sequencing method was used to detect each group of samples. Results: The resistance genes showed the following trend, from high to low relative abundance: *tetW* was the highest, followed by *tetW/N/W*, then *lnuA*; and others from high to low were *mdtB*, *lnuC*, *ANT6-la*, *ErmB*, *mdtC*, *ErmQ*, *tetBP*, *vatE*, *evgS*, *acrB*, *cpxA*, *mefA*, *Escherichia coli-ampC*, *tetL*, *yojl*, *AcrF* and *mdtA*. Conclusions: All groups administered enrofloxacin and oregano oil did not develop a drug-resistant phenotype during the 5-day treatment period, as grouped in this trial. In 2022, after Announcement No. 194 of the Ministry of Agriculture and Rural Affairs in China, the antimicrobial resistance (AMR) trend declined, but it did not fundamentally change, presumably due to the impact of environmental pollution caused by the long-term use of antimicrobials.

**Abstract:**

Background: Basic data concerning the gut microbiota of the main animal husbandry breeds (pigs and chickens) are scarce in China. The dynamics of gut microbiota (pigs and chickens) in China and antibiotic resistance genes carried by microorganisms in the natural environment are unknown. Methods: Free range and factory-farmed Gushi chickens and Huainan pigs were divided into eight groups. Faecal samples were collected from each group, and the metagenomic sequencing method was used to detect each group of samples. Results: The resistance genes showed the following trend, from high to low relative abundance: *tetW* was the highest, followed by *tetW/N/W*, then *lnuA*; and others from high to low were *mdtB*, *lnuC*, *ANT6-la*, *ErmB*, *mdtC*, *ErmQ*, *tetBP*, *vatE*, *evgS*, *acrB*, *cpxA*, *mefA*, *Escherichia coli-ampC*, *tetL*, *yojl*, *AcrF* and *mdtA*. All groups administered enrofloxacin and oregano oil did not develop a drug-resistant phenotype during the 5-day treatment period, as grouped in this trial. In 2022, after Announcement No. 194 of the Ministry of Agriculture and Rural Affairs in China, the antimicrobial resistance (AMR) trend declined, but it did not fundamentally change, presumably due to the impact of environmental pollution caused by the long-term use of antimicrobials.

## 1. Introduction

The discovery and widespread use of antimicrobials in the development of human civilization has an indelible meaning [1]. The extensive clinical application of antibiotics has greatly reduced the morbidity and mortality of bacterial infectious diseases in humans and animals [2], but due to the irregular use of antimicrobials in China (the largest producer and consumer of antimicrobials), severe bacterial resistance has emerged. Even the prevalence of cross-resistance [3] and multidrug resistance [4,5,6,7,8,9,10,11] has increased. In recent years, to reduce the irrational use of antimicrobials and control drug resistance, the government of China has introduced a series of policy measures, including the National Action Plan for Containment of Bacterial Resistance. The Ministry of Agriculture and Rural Affairs issued the National Action Plan for Restraining Bacteria of Animal Origin and the National Five-Year Action Plan for Comprehensive Management of Veterinary Drugs (Antibacterial Drugs), aimed at curbing bacterial resistance. The antimicrobial resistance (AMR) of bacteria in China’s livestock and poultry breeding industry has begun to improve.

However, in the long term, AMR has become a major threat to human health in the 21st century. The animal breeding industry is generally believed to be one of the main sources of AMR, which causes resistant bacteria and genes to circulate through the entire chain of animals, food, environment, and humans, and threatens human health. At present, AMR is common in bacteria of animal origin. Multidrug resistant and even pandrug resistant strains are constantly appearing [12,13,14,15,16], which has a huge impact on food safety and public health [17].

Irrational use of antimicrobials promotes the emergence of AMR, resulting in changes in the composition and diversity of animal gut microbiota, which adversely affect host health [18]. Quantitative data on antibiotic resistance genes (ARGs) in pigs and chickens, the main food animals in China, are still lacking, and the relationship between ARGs and bacterial species in the gut microbiome of pigs and chickens has not been elucidated [19]. For most bacteria, metagenomic sequencing technology can predict AMR with high accuracy and has become an effective tool for analysing AMR [20].

The study of microorganisms has been based mainly on pure culture for the hundreds of years since Antoni van Leeuwenhoek invented the microscope. Among the trillions of microbial species, only 0.1–1% of the species are culturable [21], which greatly limits the study of microbial diversity and molecular mechanisms of population resistance phenotypes.

Metagenomics is a method that was first proposed by Handelman [22] to study all the genomic information contained in the microbial population. Then, Kevin et al. defined metagenomics as a discipline that bypasses the isolation and culture of microbial individuals and applies genomics technology to study the microbial community in the natural environment. Metagenomics avoids the isolation and cultivation of microorganisms in the sample, provides a way to study microorganisms that cannot be isolated and cultivated, and more realistically reflects the composition and interaction of microorganisms in the sample to study their metabolic pathways and gene function at the molecular level [23].

In recent years, with the rapid development of sequencing technology and information technology, the use of next-generation sequencing technology to study metagenomics can quickly and accurately obtain a large amount of biological data and rich microbial research information [24,25,26]. Sequencing technology is an important means of characterisation that provides a powerful tool for better understanding the molecular mechanism of the microbial resistance phenotype.

According to the statistics of the World Food and Agriculture Organization, China’s number of live pigs, total pork production and poultry egg production ranks first in the world, and poultry egg production has ranked first in the world for many consecutive years. China has become one of the most important animal husbandry countries. The current understanding of the pig and chicken gut microbiome is limited, due mainly to the lack of comprehensive pig and chicken gut microbial reference gene sets and genome sets, which severely limits the mining and use of metagenomic sequencing data.

Fluoroquinolones are important antimicrobials used to treat Salmonella infections in poultry. Studies have shown that high-dose enrofloxacin (ENR, 100 mg/kg body weight (BW)) has a good killing effect on Salmonella enterica in chickens, but at the same time, the composition and structure of intestinal flora are greatly affected [27].

The observed intestinal ENR concentrations were shown to be both theoretically (based on pharmacokinetic and pharmacodynamic principles) and effectively (in vivo measurement) capable of significantly reducing the intestinal *E. coli* wild-type population [28].

In this study, the characteristics of drug resistance groups, drug-resistant bacteria and ARG contamination levels of pigs and chickens on different farms under different feeding modes were studied to provide a reference for the optimisation of antibiotic use in farms.

## 2. Methods

### 2.1. Group Test and Sample Collection

In this study, ten fresh faeces of Gushi chickens were collected from an intensive farm in Gushi County, Henan Province, P.R. China, on 15 July 2022, and ten fresh faeces of Gushi chickens were collected from a family farm. Ten fresh faeces of Huainan pigs were collected from an intensive Huainan pig farm, and 10 fresh faeces of Huainan pigs were collected from a mountain farm. The above collected subjects had not used antimicrobials in the past 2 months. On 19 July 2022, 10 faeces of the factory-bred Gushi chicken oregano oil group, 10 faeces of the enrofloxacin group (treatment amount), and 10 faeces of the factory-bred Huainan pig oregano oil group and enrofloxacin group (treatment amount) were collected, respectively. All animal-related work was carried out in strict accordance with the “Administrative Regulations on Laboratory Animals” formulated by the Ministry of Agriculture and Rural Affairs of China, and the project was approved by the Animal Ethics and Welfare Committee (AEWC) of Xinyang Agriculture and Forestry University, and then transported to the laboratory for extraction of total nucleic acid from the samples. Total DNA and total RNA were extracted from 80 samples.

Considering the consistency of animal species, age, weight, feeding place, feed and drinking water, the following test scheme was adopted in order to reduce the impact of the test on animal production. The sample size of this experiment was sufficient to reflect the basic distribution situation of the intestinal flora of the animals.

A. Free-range Gushi chicken without an additive group;
B. Factory-bred Gushi chicken oregano oil group (0.25 mL/kg BW);
C. Factory-bred Gushi chicken ENR group (5.0 mg/kg BW);
D. Free-range Huainan pigs without an additive group;
E. Factory-bred Huainan pigs without an additive group;
F. Factory-bred Huainan pigs (2021011803). All samples were stored in the sample preservation solution, mixed with pig oregano oil group (0.25 mL/kg BW);
G. Factory-bred Huainan pigs ENR Group (5.0 mg/kg BW)
H. Factory-bred Gushi chicken without an additive group.

Factory or intensive breeding was performed by industrial production in a factory farm in the following manner. First, the scale of factory breeding was large (>10,000). Second, the hybrid combination or mating line of animals with a consistent body shape, appearance, and balanced growth, were selected. Third, some mechanised and automatic equipment, such as automatic drinking fountains, automatic feeding troughs, and automatic dung cleaning, were used. Fourth, fewer staff, less land occupation, and high labor productivity, occurred. Fifth, scientific operation and management methods were used to organise production, so that production conditions and technological processes operated in accordance with standards and rules, including full price compound feed, modern epidemic prevention measures and all inflow and all outflow production process.

The family farm, or mountain farm, operated under the free-range breeding model. The family farm or mountain farm engaged in animal husbandry with an annual production of more than 500 pigs, more than 5000 chickens for broilers, more than 2000 laying hens, or more than 3000 mixed breeding poultry. The animals moved freely. The animals were fed full price compound feed, and wild and green feed. Normal epidemic prevention and expulsion of parasites occurred.

The above animals were all adult animals. Pigs were in the fattening stage (5–8 months), and chickens were in the laying stage (19–28 weeks). The ENR group and oregano oil group were administered oregano orally twice a day for 5 days. The water intake of the animals was controlled in the experiment.

The raw samples or the extracted nucleic acid samples was transported cold (in dry ice, −78.5 ℃) to the biotech company. The received samples were tested for eligibility. Qualified DNA samples were detected, and library construction and library detection were performed. Illumina NOVASEQ 6000 (Illumina, Inc., San Diego, CA, U.S.A.) was used to sequence the qualified library, and the raw data obtained by sequencing were used for later information analysis.

### 2.2. DNA Sample Detection

Library construction and sequencing yielded a sufficient amount of high-quality nucleic acids. The detection of DNA samples mainly included the analysis of DNA purity and integrity by agarose gel electrophoresis (AGE), and Qubit accurately quantified the DNA concentration.

### 2.3. Library Construction and Library Inspection

Qualified DNA samples were randomly broken into fragments of approximately 350 bp in length with a Covaris ultrasonic breaker, and the entire library was prepared through the steps of end repair, A-tail addition, sequencing adapter addition, purification, and PCR amplification.

After the library was constructed, Qubit 2.0 was used for preliminary quantification, the library was diluted to 2 ng/µL, and then an Agilent 2100 (Agilent, Santa Clara, CA, U.S.A.) was used to detect the insert size of the library. After the insert size met expectations, the Q-PCR method was used to determine the effective concentration of the library. Accurate quantification (library effective concentration > 3 nM) was performed to ensure library quality.

### 2.4. On-Board Sequencing

After the library was qualified, the different libraries were pooled according to the requirements of effective concentration and target data volume, and then Illumina NOVASEQ 6000 sequencing was performed.

### 2.5. Information Analysis Process

#### 2.5.1. Data Quality Control

There was a certain proportion of low-quality data in the raw data obtained by sequencing. To ensure the accuracy and reliability of subsequent information analysis results, quality control and host filtering of the raw data were first carried out to obtain valid data.

Reads that contained low-quality bases (quality value ≤ 38) that exceeded a certain percentage (set to 40 bp by default) were removed.

Reads with a certain proportion of N bases (default was 10 bp) were removed.

Reads whose overlap with the adapter exceeded a certain threshold (set to 15 bp by default) were removed.

#### 2.5.2. Metagenome Assembly

Metagenome assembly started from clean data after the quality control of each sample was performed.

After preprocessing, clean data were obtained, and MEGAHIT assembly software was used for assembly analysis.

The assembly parameters were: presets meta-large.

The assembled scaffolds were interrupted from N junctions to obtain sequence fragments without N, called scaftigs (i.e., continuous sequences within scaffolds).

After quality control, the clean data of each sample were compared with the assembled scaftigs of each sample using Bowtie2 Version 2.4.5 software.

The alignment parameters were: –end-to-end, –sensitive, -I 200, -X 400.

For scaftigs generated by single-sample assembly, fragments below 500 bp were filtered out, and statistical analysis and subsequent gene prediction were performed.

#### 2.5.3. Gene Prediction

Gene prediction started from scaftigs assembled from single samples. MetaGeneMark was used for gene prediction, and the genes predicted from each sample were combined to remove redundancy and construct a gene catalogue. Based on the clean data of each sample, the abundance information of the gene catalogue in each sample was obtained.

The basic steps of gene prediction and abundance analysis were as follows.

Starting from the scaftigs (≥500 bp) of each sample, MetaGeneMark was used to perform an open reading frame (ORF) prediction, and information of length less than 100 nt was filtered out.

The open reading frame (ORF) prediction results assembled by each sample were made de-redundant using CD-HIT software to obtain a nonredundant initial gene catalogue (here, in operation, the nucleic acid sequences encoded by nonredundant continuous genes are called genes). The default was identity 95%, coverage 90% for clustering, and the longest sequence was selected as the representative sequence.

The parameters were: -c 0.95, -G 0, -aS 0.9, -g 1, -d 0.

The clean data of each sample were compared with the initial gene catalogue by Bowtie2, and the number of reads of the gene in each sample was calculated.

The alignment parameters were: –end-to-end, –sensitive, -I 200, -X 400.

The genes that supported the number of reads ≤2 in each sample were filtered out, and the final gene catalogue (unigenes) for subsequent analysis was obtained.

Based on the number of reads and gene length in the alignment, the abundance information of each gene in each sample was calculated, and the calculation formula was as follows:

$$G_{k}=\frac{r_{k}}{L_{k}}\times \frac{1}{\sum_{i=1}^{n}\frac{r_{i}}{L_{i}}}$$

where *r* is the number of reads of the gene in the alignment, and *L* is the length of the gene.

Based on the abundance information of each gene in each sample in the gene catalogue, the basic information statistics, core–pan gene analysis, correlation analysis between samples, and Venn diagram analysis of the number of genes were performed.

#### 2.5.4. Species Annotation

The species annotation information of each gene (Unigene) was obtained from the gene catalogue and compared with the MicroNR library, and the species abundance table of different taxonomic levels was obtained by combining the gene abundance table.

Based on the species abundance table and functional abundance table, the abundance clustering analysis, PCA and NMDS dimensionality reduction analysis, ANOSIM analysis and sample clustering analysis were able to be performed. When group information was available, multivariate statistical analysis of Metastat and LEfSe and comparative analysis of metabolic pathways were also able to be performed to explore the differences in species composition and functional composition among samples.

#### 2.5.5. Resistance Gene Annotation

The gene catalogue and the Comprehensive Antibiotic Resistance Database (CARD) were used for annotation, the abundance distribution of resistance genes was obtained, and the species attribution and resistance mechanism of these resistance genes were found.

## 3. Results

### 3.1. Sequencing Data Preprocessing Results

**Figure 1:**
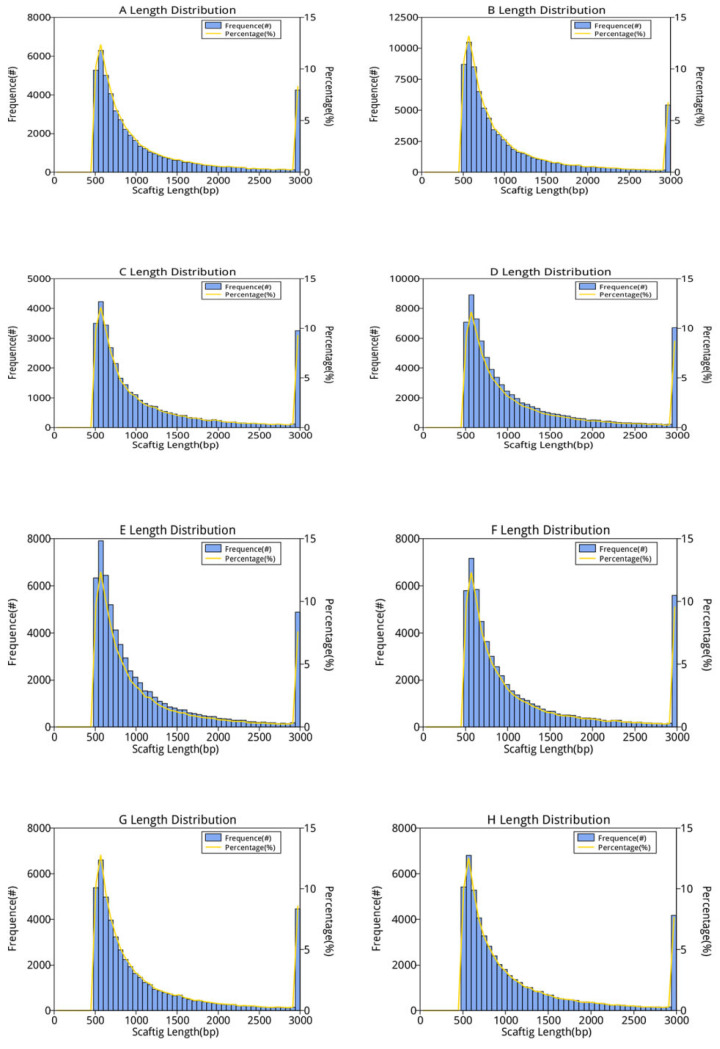
Scaftig length distribution statistics of each sample (≥500 bp). The first vertical axis (Frequence (#)) represents the number of scaftigs; the second vertical axis (Percentage (%)) represents the percentage of the number of scaftigs; the horizontal axis represents the length of scaftigs.

The sequencing data preprocessing results are shown in Table 1, Table 2 and Table 3 and Figure 1.

### 3.2. Metagenome Assembly (≥500 bp)

Based on the assembly results, the distribution of the length of scaftigs in each sample was counted and plotted, and the results are shown in Figure 1.

**Figure 2:**
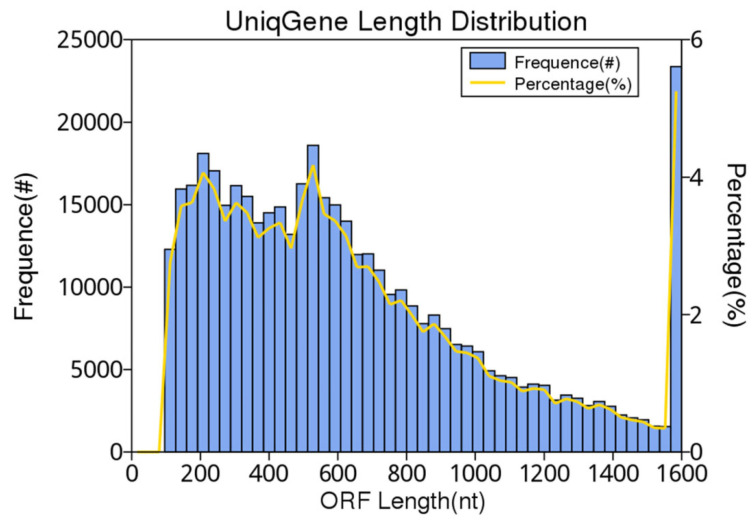
Gene catalogue length distribution. The first vertical axis (Frequence (#)) represents the number of scaftigs; the second vertical axis (Percentage (%)) represents the percentage of the number of scaftigs; the horizontal axis represents the length of scaftigs.

The gene catalogue length distribution is shown in Figure 2.

### 3.3. Core–Pan Gene Analysis

**Figure 3:**
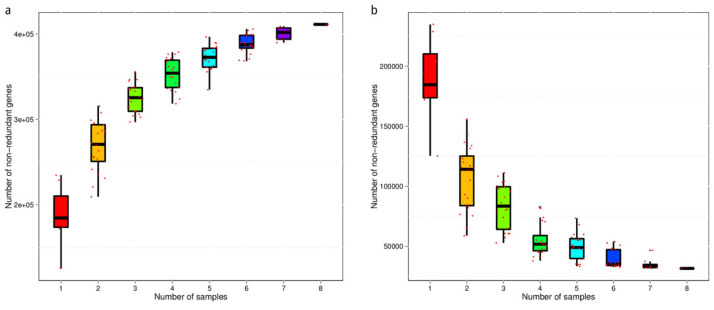
Dilution curve of the core and pan genes. (**a**) Pan gene dilution curve. (**b**) Core gene dilution curve. The abscissa indicates the number of samples. The vertical axis represents the number of genes in the samples (1–8: A–G). 4E+5: 4 × 10^5^, Same with the rest.

Based on the abundance table of genes in each sample, the number of genes in each sample can be obtained. By randomly extracting different numbers of samples, the number of genes between combinations of different numbers of samples can be obtained. Therefore, we constructed and mapped the dilution curve of the core and pan genes, and the results are shown in Figure 3.

**Figure 4:**
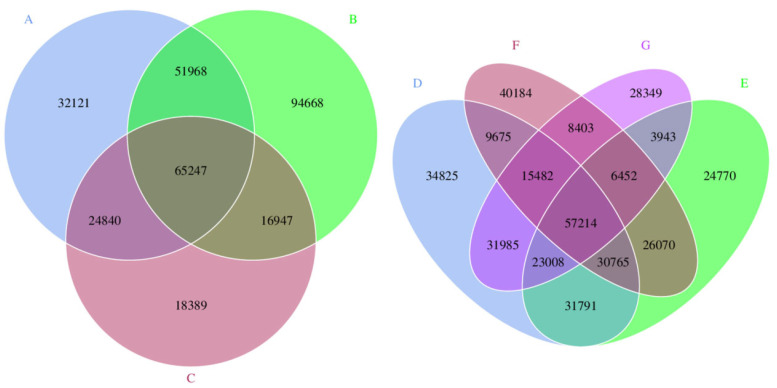
Number of genes. In the figure, each circle represents a sample; the numbers in the overlapping parts of the circles represent the number of genes shared between the samples; the numbers without the overlapping part represent the unique number of genes of the sample.

To examine the number of genes between the specified groups, the coexistence and unique information between different groups were analysed, and a Venn graph, or petals, were drawn. The results are shown in Figure 4.

### 3.4. Gene Number-Based Correlation Analysis between Samples

**Figure 5:**
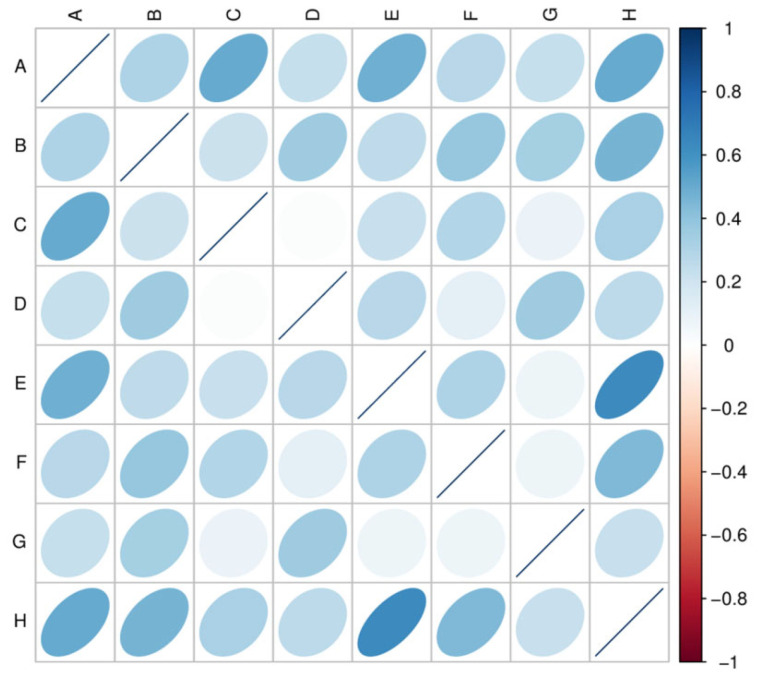
Heatmap of the correlation coefficient between samples. In the figure, different colours represent the Spearman correlation coefficient; see the legend on the right for the relationship between the correlation coefficient and colour. The darker the colour is, the greater the absolute value of the correlation coefficient between samples. The right deviation of the ellipse indicates that the correlation coefficient is positive and the left deviation is negative. The flatter the ellipse, the larger the absolute value of the correlation coefficient.

Biological repetition is necessary for biological experiments, and high-throughput sequencing technology is no exception. The correlation between the genetic abundance of the sample is an important indicator for testing the reliability of the experiment and whether the sample is selected. The closer to the correlation coefficient, the higher the similarities of the gene abatement mode between the samples. The results are shown in Figure 5.

### 3.5. Microbial Species Annotation

#### 3.5.1. Basic Steps for Species Annotation

Genes were compared in various functional databases by DIAMOND software. Unigenes were compared with sequences for alignment (basic local alignment search tool (BLAST)p, e-value ≤ 1 × 10^−5^).

Alignment result filtering: for the alignment result of each sequence, the alignment result with evalue ≤ minimum evalue*10 was selected for subsequent analysis.

After filtering, since each sequence may have multiple alignment results and obtain multiple different species classification, to ensure its biological significance, the lowest common ancestor (LCA) algorithm (applied to the systematic classification of MEGAN software) was applied.

The taxonomic level before a branch represented the species annotation information of the sequence.

Based on the LCA annotation results and gene abundance table, the abundance information of each sample at each taxonomic level (Jiemenae, genus and species) was obtained. The abundance of a species in a sample was equal to the sum of gene abundances that the species annotated as the species.

Based on the LCA annotation results and the gene abundance table, the number of genes in each sample at each taxonomic level (Kingmenae, genus and species) was obtained. Among the genes of the species, the number of genes whose abundance was not 0 was presented.

Based on the abundance table at each taxonomic level (Jiemenae genus and species) and Krona analysis, the relative abundance overview was displayed, the abundance cluster heatmap was displayed, PCA and nonmetric multidimensional scaling (NMDS) dimensionality reduction were analysed, the ANOSIM difference between (within) groups was analysed, and Metastat and LEfSe multivariate statistical analyses of different species between groups were performed.

#### 3.5.2. Overview of the Relative Abundance of Species

**Figure 6:**
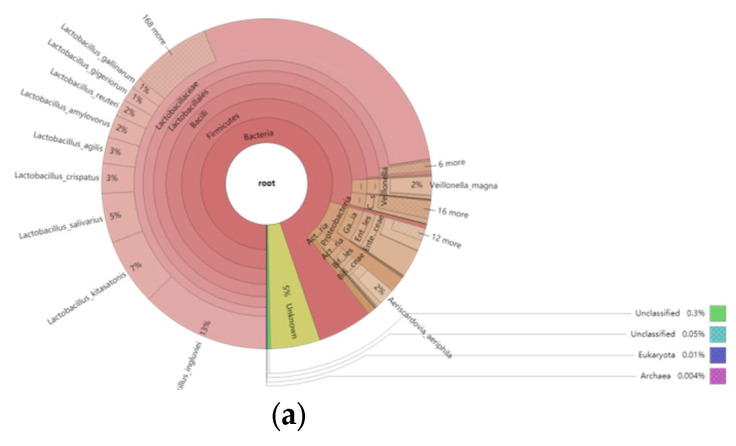

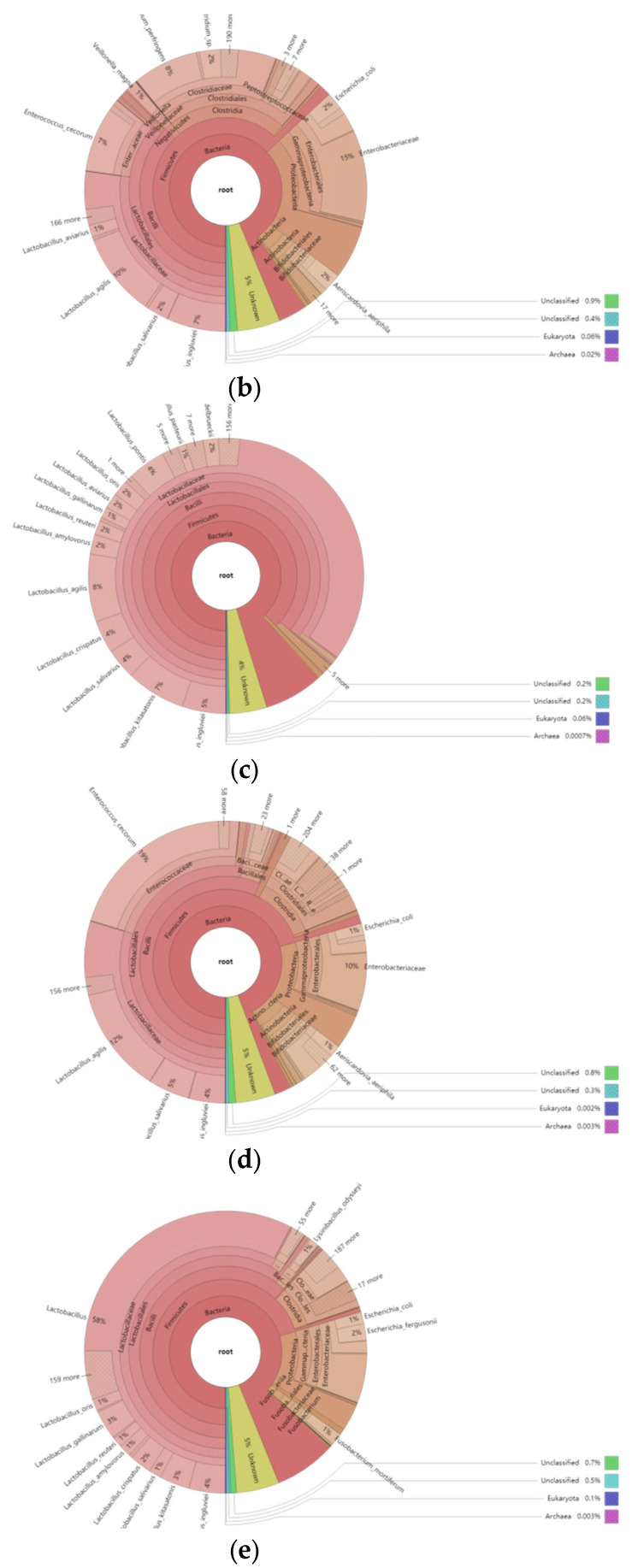

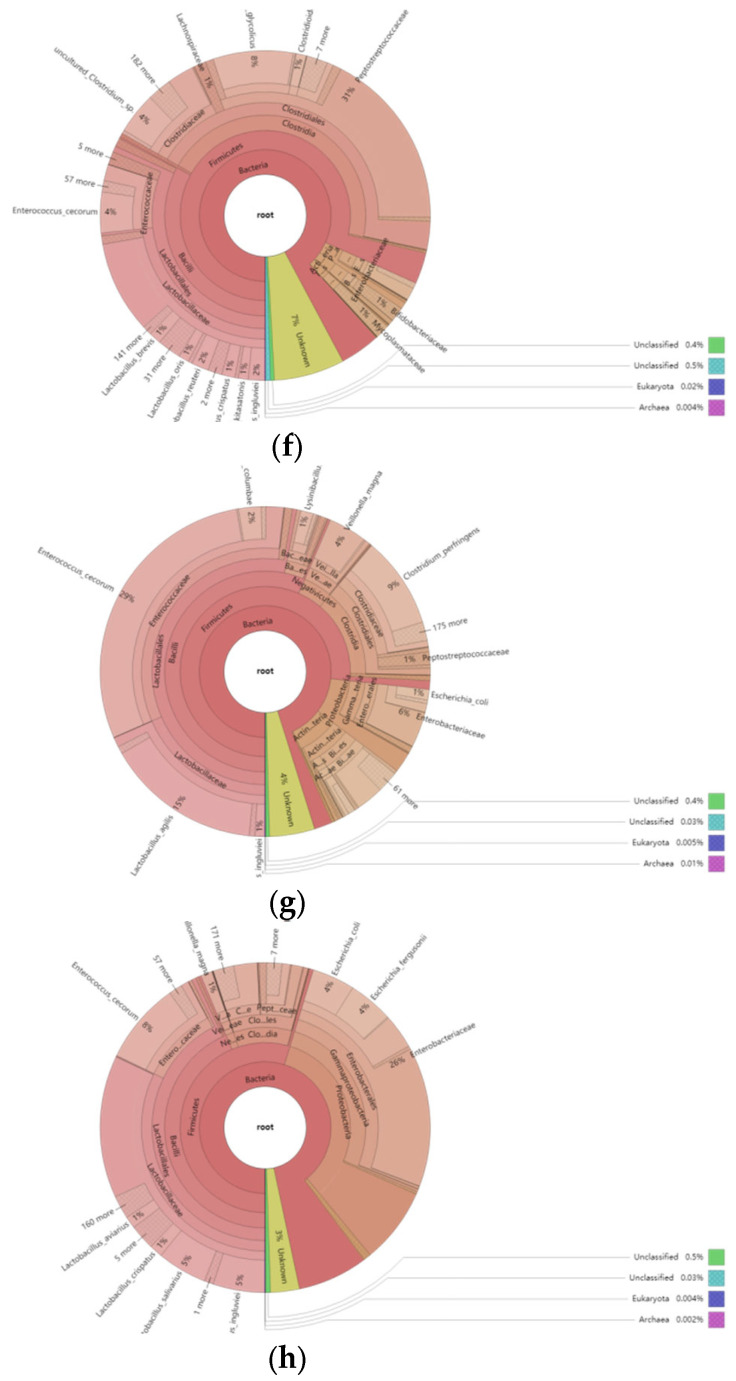
(**a**–**h**). Annotate results for species using Krona. In Figure 6, the circles represent different taxonomic levels (kingdom, phylum, class, order, family, genus, species) from inside to outside; the size of the sector represents the relative proportion of different species.

To comprehensively and intuitively display the relative abundance of different classification levels in each sample, we used Krona to display the species annotation results. The results are shown in Figure 6.

Studies have shown that the gut microbiota is individual-specific, and the composition of the microbiota of pigs under different feeding conditions and different intestinal parts is different.

**Figure 7:**
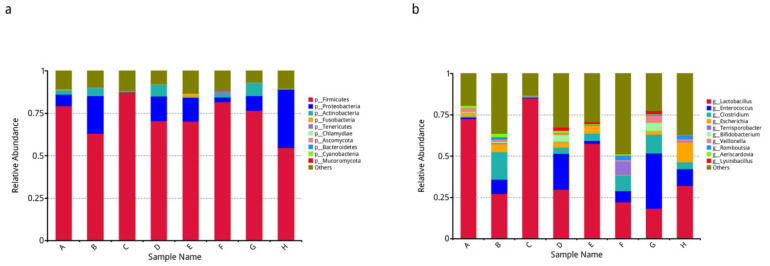
The relative abundance of species was presented at the phylum and genus levels. (**a**) Histogram of relative abundance was shown at the gate level. (**b**) Histogram of relative abundance was presented at the genus level. The horizontal axis represents the sample name. The vertical axis represents the relative proportion of species annotated to a certain type. The legend on the right is the species category corresponding to each colour block.

Based on the relative abundance tables of different classification levels, the top 10 species with the largest relative abundance were selected in each group, the rest of the species were set as Others, and the species annotation results corresponding to each sample in the column chart of relative abundance at different taxonomic levels are shown in Figure 7.

#### 3.5.3. Cluster Analysis of the Number and Relative Abundance of Annotated Genes

**Figure 8:**
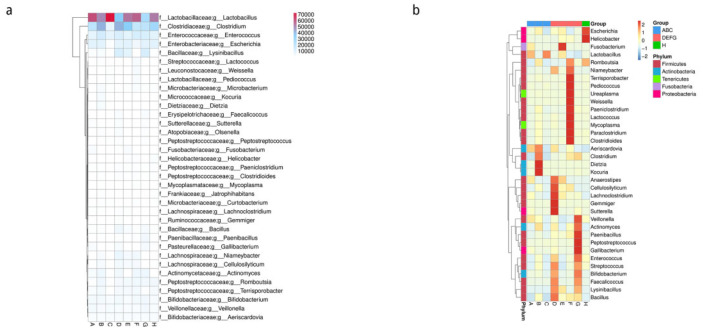
Cluster heatmap of the number and abundance of genes at the genus level. (**a**) Unannotated number statistical heatmap; the horizontal axis is the sample name; the vertical axis is the species information; and different colours represent the number of unigenes. (**b**) Cluster heatmap of relative abundance at the genus level; the horizontal direction is the sample information; the vertical direction is the species information. The cluster tree on the left in the figure is a species cluster tree. The value corresponding to the intermediate heatmap is the Z-value obtained after the relative abundance of species in each row is normalised; that is, the Z-value of a sample in a certain classification is the value obtained by dividing the difference between the relative abundance of the sample in that classification and the average relative abundance of all samples in that classification by the standard deviation of all samples in that classification.

Based on the relative abundance tables of different classification levels, the top 35 genera in terms of abundance and their abundance information in each sample were selected to draw a heatmap, and were clustered at the species level to facilitate the display of results and information discovery to determine the species with more aggregation in the sample. The results are shown in Figure 8.

#### 3.5.4. Dimension Reduction Analysis Based on Species Abundance

**Figure 9:**
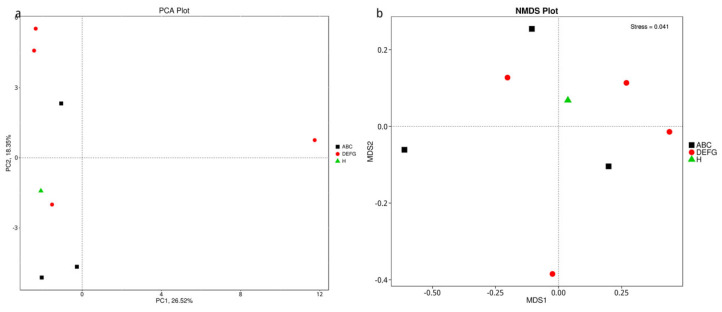
PCA and NMDS results of species based on the phylum level. (**a**) For phylum–level PCA, the abscissa represents the first principal component, and the percentage represents the contribution value of the first principal component to the sample difference. The ordinate represents the second principal component, and the percentage represents the contribution value of the second principal component to the sample difference. Each point in the figure represents a sample, and the samples in the same group are represented by the same colour. (**b**) For gate-level NMDS analysis, each point in the figure represents a sample, the distance between points represents the degree of difference, and the samples in the same group are represented by the same colour. When the stress is less than 0.2, NMDS analysis has a certain reliability.

At present, principal component analysis (PCA) and nonmetric multidimensional scaling (NMDS) are the main methods of dimension reduction analysis applied to ecological research. Among these methods, PCA is a dimension reduction analysis based on a linear model that applies the method of variance decomposition to reduce the dimension of multidimensional data to extract the most important elements and structures in the data. PCA can extract two coordinate axes that reflect the differences between samples to the greatest extent to reflect the differences of multidimensional data on the two-dimensional coordinate map and then reveal the simple laws in the complex data background. NMDS is a nonlinear model whose purpose is to overcome the shortcomings of linear models and better reflect the nonlinear structure of ecological data. NMDS analysis is applied to reflect the species information contained in the sample in the form of points in the multidimensional space, while the difference between different points is reflected by the distance between points, which can reflect the differences between groups or within groups of samples. Based on the species abundance tables at different classification levels, we conducted PCA and NMDS analysis. If the species composition of the samples was more similar, then the distance between them was closer, as shown in the PCA and NMDS diagrams in Figure 9.

#### 3.5.5. Dimension Reduction Analysis of Bray–Curtis Distance Based on Species Abundance

**Figure 10:**
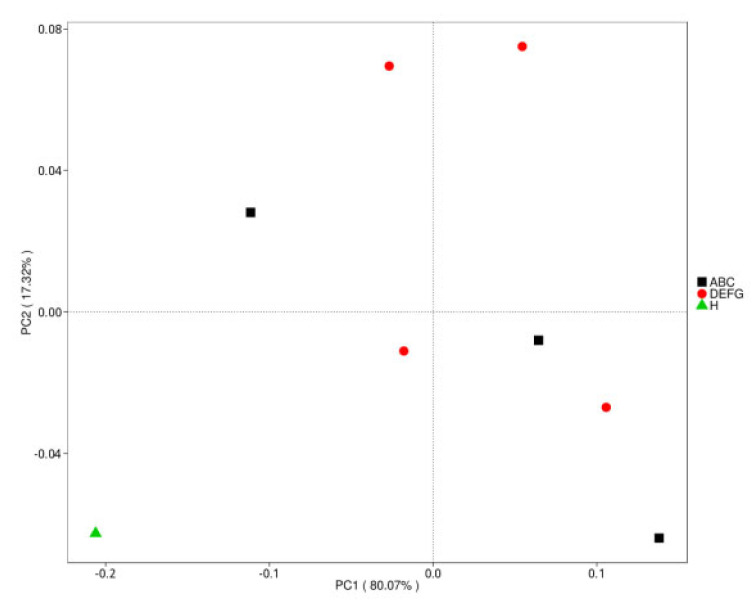
Species PCoA results based on the phylum level. Note: the abscissa represents one principal component, the ordinate represents another principal component, and the percentage represents the contribution value of the principal component to the sample difference. Each point in the figure represents a sample, and the samples in the same group are represented by the same colour.

Principal coordinate analysis (PCoA) extracts the most important elements and structures from multidimensional data through a series of eigenvalues and eigenvector ordering. We conducted PCoA based on the Bray–Curtis distance and selected the principal coordinate combination with the largest contribution rate for graphic display. If the sample distance was closer, the species composition structure was more similar. Therefore, the samples with high similarity in community structure tended to gather together, and the samples with large differences in community were far apart. The Bray–Curtis distance matrix was obtained based on species abundance tables at different taxonomic levels. The results of the PCoA analysis based on the gate level are shown in Figure 10.

### 3.6. Cluster Analysis of Samples Based on Species Abundance

**Figure 11:**
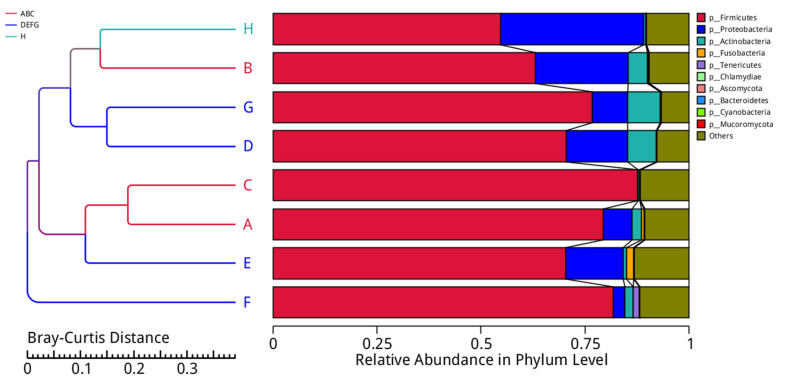
Clustering tree based on the Bray–Curtis distance. The left side is the Bray–Curtis distance clustering tree structure; the right side is the species relative abundance distribution map of each sample at the phylum level.

To study the similarity of different samples, a clustering tree of the samples was constructed by clustering the samples. The Bray–Curtis distance is the most common distance indicator used in systematic clustering. It is used mainly to describe the similarity between samples, and its size is the main basis for sample classification. Starting from the abundance table of genes in each sample, the Bray–Curtis distance matrix was used to perform cluster analysis among samples, and the clustering results were integrated with the relative abundance of species at the phylum level in each sample for display. The results are shown in Figure 11.

### 3.7. Linear Discriminant Analysis Effect Size (LEfSe) Analysis of Different Species between Groups

**Figure 12:**
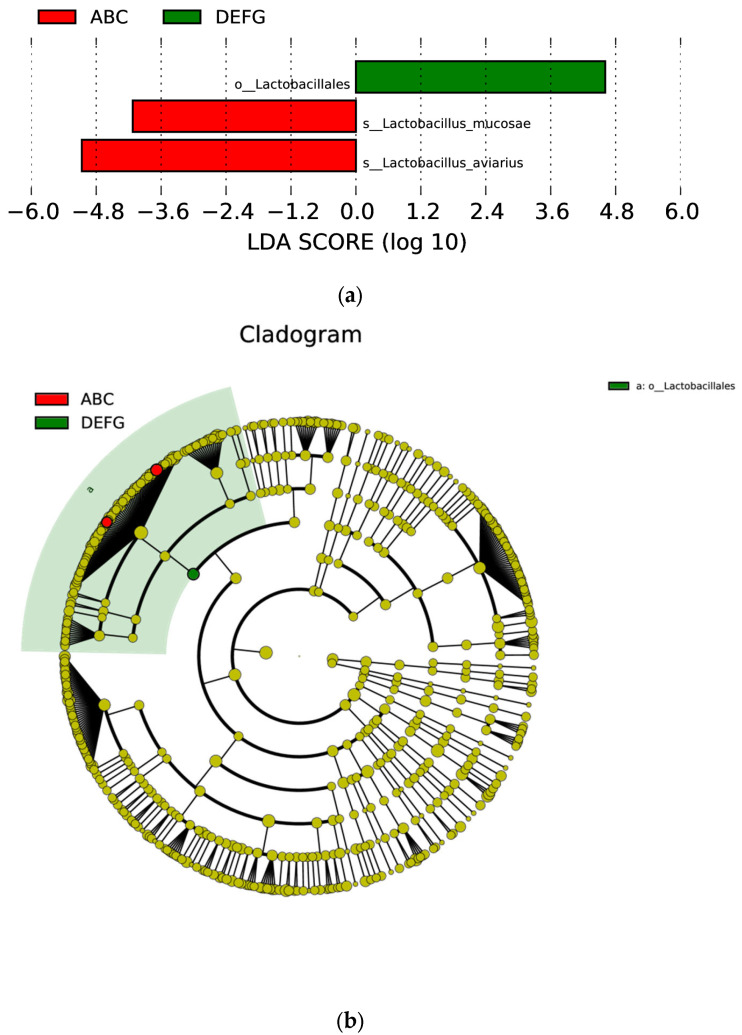
Distribution of LDA values and evolutionary clades of different species. (**a**) is the LDA value distribution of different species. The LDA value distribution histogram shows the species whose LDA score is greater than the set value (the default setting is 4), that is, the biomarker with a significant difference between groups, and the length of the histogram represents the impact size of different species (i.e., LDA score). (**b**) shows the evolutionary clades of different species. The circles radiating from the inside to the outside represent the taxonomic level from phylum to genus (or species). Each small circle at a different taxonomic level represents a taxonomy at that level, and the diameter of the small circle is proportional to the relative abundance. Colouring principle: Species with no significant difference are uniformly coloured yellow, and the biomarkers of different species are coloured according to the group. The red nodes represent the microbial groups that play an important role in the red group, and the green nodes represent the microbial groups that play an important role in the green group. The microorganism species names represented by the English letters in the figure are shown in the legend on the right.

To screen the species biomarkers with significant differences between groups, first, the rank sum test method was used to detect the different species between different groups, and linear discriminant analysis (LDA) was used to achieve dimensionality reduction and evaluate the influence of the different species; that is, the LDA score was obtained. The LEfSe analysis results of species with differences between them included three parts, namely, the LDA value distribution histogram, the evolutionary branch diagram (phylogenetic distribution), and the abundance comparison diagram of biomarkers with significant differences between groups. The LDA value distribution map and evolutionary branch map of different species are shown in Figure 12.

### 3.8. Annotation of Resistance Genes

Resistance genes are ubiquitous in both human intestinal microorganisms and other environmental microorganisms. The abuse of antimicrobials leads to irreversible changes in the microbial community in the human body and the environment, which poses risks to human health and the ecological environment. Therefore, research on resistance genes has received extensive attention from researchers. CARD is a resistance gene database that has emerged in recent years. It has the advantages of comprehensive information, user-friendliness, timely updates and maintenance. The core component of this database is antibiotic resistance ontology (ARO), which integrates information such as sequence, antibiotic resistance, mechanism of action and association between AROs and provides online interfaces between ARO and databases such as Protein Data Bank (PDB) and the National Center for Biotechnology Information (NCBI).

#### 3.8.1. Basic Steps of Resistance Gene Annotation

Unigenes were compared with the CARD database using the resistance gene identifier (RGI) software provided by the CARD database (v2.0.1) (RGI has built-in BLASTp, and the results were scored by bitscore value comparison).

According to the comparison results of RGI and the abundance information of unigenes, the relative abundances of AROs were calculated.

Based on the abundance of ARO, the following were generated: the column diagram of abundance, the heatmap of abundance clustering, the circle diagram of abundance distribution, the analysis of ARO differences between groups, and the species attribution analysis of resistance genes (unigenes annotated to ARO) (for some AROs with longer names, we will display them in the form of the first three words and underlined abbreviations).

#### 3.8.2. Overview of Resistance Gene Abundance

**Figure 13:**
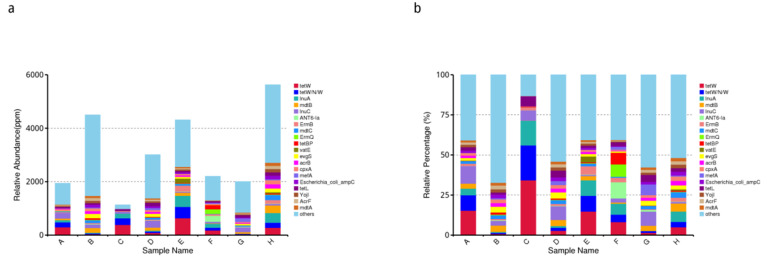
Histogram of the abundance of different AROs in each sample. (**a**) The relative abundance of all genes of ARO in each sample, in ppm, which is the result of enlarging the original relative abundance data by 106 times. (**b**) Indicates the relative abundance of the top 20 AROs among all AROs, and the others are the sums of the relative abundances of the top 20 non-AROs.

Based on the relative abundance table of resistance genes, we calculated the content and percentage of ARO in each sample and screened the top 20 AROs with the largest abundance. The results are shown in Figure 13.

**Figure 14:**
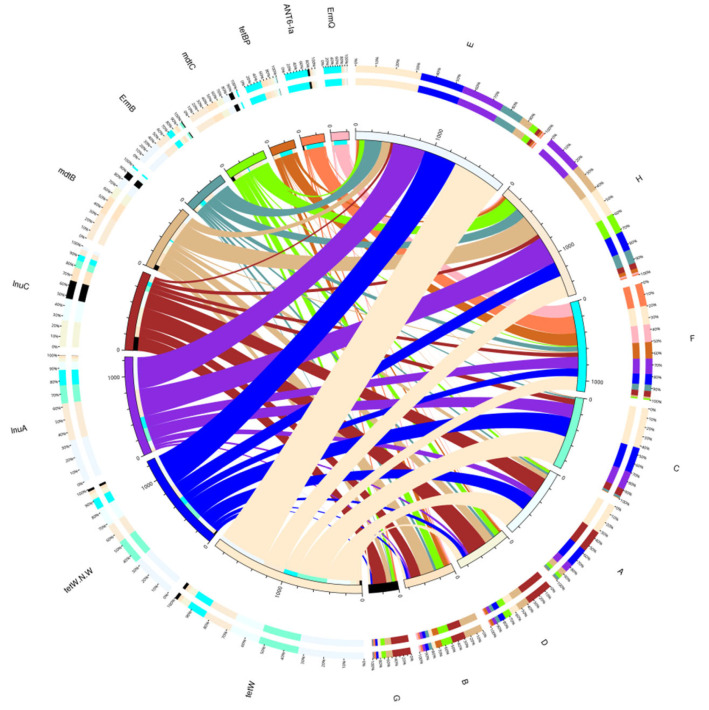
Overview circle of resistance genes. The circle diagram is divided into two parts, with sample information on the right and ARO information on the left; Different colours in the inner circle represent different samples and AROs. The scale is the relative abundance, in ppm. The left side is the sum of the relative abundances of each ARO in a sample, and the right side is the sum of the relative abundances of each ARO in a sample. The left side of the outer ring is the relative percentage of each sample in an ARO, and the right side of the outer ring is the relative percentage of each ARO in a sample.

To more intuitively observe the abundance ratio of ARO in each sample as a whole, and more intuitively display the overall distribution of ARO abundance, the ARO with the largest abundance of TOP10 was selected to be drawn as an overview circle, as shown in Figure 14.

**Figure 15:**
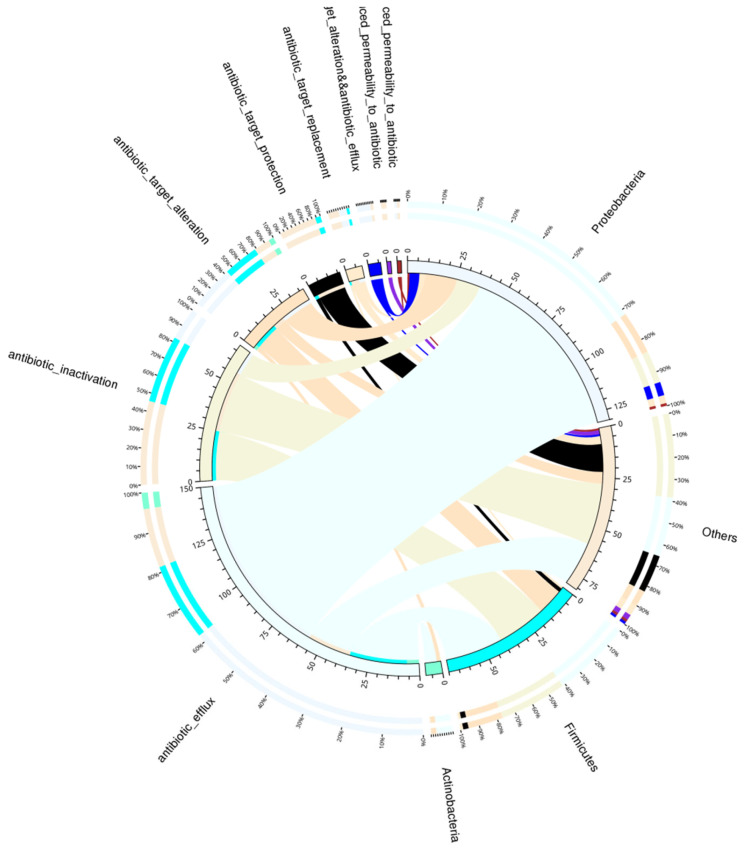
Overview circle of resistance mechanism and species. The circle diagram is divided into two parts: the right side is phylum-level species information, and the left side is resistance-mechanism information. Different colours in the inner circle indicate the resistance mechanisms of different species and resistances, and the scale is the number of genes. The left side is the sum of the number of resistance genes containing such resistance mechanisms in the species, and the right side is the sum of the number of resistance genes contained in the species in different resistance mechanisms. The left side of the outer circle is the relative proportion of resistance genes in each species to the resistance genes of their resistance mechanisms, and the right side of the outer circle is the relative proportion of resistance genes in each resistance mechanism to the resistance genes of their species.

The resistance mechanism of resistance genes in the CARD database was classified, and the following resistance mechanism distribution map was drawn according to the relationship between the action mechanism of these resistance genes and species. The results are shown in Figure 15.

The sequencing results clearly showed that the abundance and relative percentage of drug resistance genes in the eight typical samples showed a trend from high to low.

The relative abundance and relative percentage of drug-resistant genes showed the following trend of descending order: *tetW*, *tetW/N/W*, *lnuA*, *mdtB*, *lnuC*, *ANT6-la*, *ErmB*, *mdtC*, *ErmQ*, *tetBP*, *vatE*, *evgS*, *acrB*, *cpxA*, *mefA*, *Escherichia coli-ampc*, *tetL*, *yojl*, *AcrF* and *mdtA*.

All groups administered enrofloxacin and oregano oil did not develop a drug-resistant phenotype during the 5-day treatment period, as grouped in this trial.

## 4. Discussion

One of the most important functions of the gut microbiota is to prevent bacterial overgrowth and infection by pathogenic microorganisms, mainly through the production of antibacterial substances, site-occupying protection, and competition for nutrients with pathogenic bacteria.

Bacteriocins are antimicrobial peptides or complex proteins synthesised by the ribosomes of various Gram-positive and Gram-negative bacteria, and have bacteriostatic and bactericidal effects. For example, the bacteriocin-like substances secreted by lactic acid bacteria have broad-spectrum antibacterial effects and can inhibit the growth and reproduction of Gram-negative bacteria such as *Escherichia coli*. In addition, metabolites such as short-chain fatty acids (SCFAs) produced during bacterial metabolism can reduce intestinal pH, thereby inhibiting the growth and reproduction of exogenous bacteria. At the same time, the acidification of the intestinal environment promotes the acceleration of intestinal peristalsis, resulting in exogenous bacteria not interacting with the mucosa, and exogenous bacteria are excreted. The space-occupying protective effect of intestinal flora is to form a biofilm by closely combining with intestinal mucosal epithelial cells, thereby effectively preventing pathogenic bacteria from contacting the intestinal mucosa. Under the limited nutrient intake of the host, intestinal flora, especially symbiotic bacteria, have a natural competitive advantage for these nutrients, inhibiting the growth and reproduction of pathogenic bacteria.

The diversity and distribution of drug resistance groups in pig and chicken faeces under different feeding modes (free range and factory feeding) were systematically analysed.

Based on the basic situation of the intestinal flora of animals (pig and chicken) in central China, the drug-resistant phenotype presents dual traces of history and current practice. Tetracycline resistance remains the most abundant type of resistance, and this result is consistent with the findings of other Chinese scholars in other provinces [29,30].

Some factory-farmed pigs and chickens have higher levels of intestinal bacterial resistance than free-range pigs and chickens.

In this study, an integrated gene set of gut microbes was constructed using the metagenomic sequencing data of eight groups of swine and chicken gut microbes from different feeding patterns, combined with the gene sets of existing metagenomic sequencing data, which included 237,785 complete genes, of which 5% were unknown, expanding the comprehensiveness and completeness of the swine and chicken gut microbial gene sets. The genus-level dominant species represented by the 35 metagenomic assembled genomes were analysed by software. Using analytical software, we further revealed the diversity of swine and chicken gut microbiota composition and drug resistance phenotypes and identified differences in drug resistance phenotypes.

Comparative studies in animal samples provide important links to understanding the global distribution of ARGs and the spread of multidrug-resistant bacteria, resistance exchange networks, and how different habitats and phylogenetic relationships influence the evolutionary dynamics of global antimicrobial resistance.

Drug resistance in pathogenic bacteria is a growing threat to global health, and the development and spread of drug resistance in microorganisms is largely attributable to the misuse of antimicrobials.

AMR in animals is country-, or even, region-specific. A map of the global distribution of resistance shows resistance hotspots in northeastern China, northeastern India and northern Pakistan, while resistance is just beginning to emerge in central India and central and southern China, Kenya, Uruguay and southern Brazil [31,32,33,34]. In 2022, after the announcement of the Ministry of Agriculture and Rural Affairs No. 194, this trend declined, but it did not fundamentally change, presumably due to the impact of environmental pollution caused by the long-term use of antimicrobials.

There are differences in the intestinal microbiota under different feeding styles [35]. Animals receive different nutrients from free-range and factory-raised feed, which also has an impact on their gut flora. After group administration, the abundance of intestinal flora of animals in the two feeding modes showed same trend, and the abundance of AMR genes had their own characteristics, which may be related to the long-term drug history, the AMR in the environment, and the feed of adult animals.

## 5. Conclusions

Metagenomic sequencing was conducted to study the relative changes of intestinal microflora and drug resistance genes in pigs and chickens in central China, after treatment amounts of enrofloxacin and oregano oil were added into the feed in factory and free range mode, respectively. The consistency of the trend of intestinal microflora changes and the existence of drug resistance genes were found after treatment, providing reference for rational drug use in the veterinary clinic. The results showed that *tetW* was the highest relative abundance resistance gene, followed by *tetW/N/W*, then *lnuA*; and others from high to low were *mdtB*, *lnuC*, *ANT6-la*, *ErmB*, *mdtC*, *ErmQ*, *tetBP*, *vatE*, *evgS*, *acrB*, *cpxA*, *mefA*, *Escherichia coli-ampC*, *tetL*, *yojl*, *AcrF* and *mdtA*.

## Figures and Tables

**Table 1:** Data preprocessing statistics table.

#Sample	InsertSize (bp)	RawData	CleanData	Clean_Q20	Clean_Q30	Clean_GC (%)	Effective (%)
A	350	6633.18	6629.30	96.2	89.95	40.72	99.941
B	350	6982.84	6969.06	96.11	89.8	41.56	99.803
C	350	6714.52	6708.95	96.27	90.09	40.08	99.917
D	350	6155.96	6152.45	96.03	89.66	41.1	99.943
E	350	6822.24	6818.75	95.88	89.34	39.31	99.949
F	350	6528.38	6524.90	95.99	89.38	35.21	99.947
G	350	6884.02	6881.10	95.95	89.5	39.52	99.958
H	350	6645.17	6639.25	96.39	90.44	42.14	99.911

#Sample, sample name; InsertSize (bp), insert fragment length (default 350 bp library); RawData, the raw data of the computer; CleanData, the valid data obtained and filtered; Clean_Q20, percentage of bases with a sequencing error rate of less than 0.01 (quality value greater than 20) in CleanData; Clean_Q30, percentage of bases in CleanData with a sequencing error rate less than 0.001 (quality value greater than 30); Clean_GC (%), GC content of bases in CleanData; Effective (%), the percentage of effective data (CleanData) to raw data (RawData).

**Table 2:** The assembly results of each sample. Scaftig information (≥500 bp).

Sample ID	Total Len. (bp)	No.	Average Len. (bp)	N50 Len. (bp)	N90 Len. (bp)	Max Len. (bp)
A	77,097,982	50,858	1515.95	2087	625	304,611
B	103,806,670	79,760	1301.49	1565	598	94,301
C	53,150,756	34,873	1524.12	2127	628	182,450
D	114,767,070	76,524	1499.75	2000	634	269,282
E	91,187,139	64,129	1421.93	1804	619	395,129
F	98,440,700	58,234	1690.43	2689	644	185,631
G	85,667,957	51,678	1657.73	2510	637	297,861
H	77,519,231	54,238	1429.24	1830	617	157,857

Sample ID, indicates the sample name; Total Len. (bp), indicates the total length of the assembled scaftigs; No., indicates the total number of scaftigs assembled; Average Len. (bp), indicates the average length of scaftigs; N50 Len. (bp), indicates that the scaftigs were sorted by length and then summed from long to short. When the sum value reached 50% of the total length of scaftigs, the length value of scaftigs was found; N90 Len. (bp), indicates that the scaftigs are sorted by length and then summed from long to short. When the sum value reaches 90% of the total length of scaftigs, the length value of scaftigs was found; Max Len, indicates the length of the longest scaftigs assembled.

**Table 3:** Gene catalogue basic information. ORFs No. indicates the number of genes in the gene catalogue.

ORFs No.	445,260
integrity:end	82,047 (18.43%)
integrity:start	93,479 (20.99%)
integrity:all	237,785 (53.4%)
integrity:none	31,949 (7.18%)
Total Len. (Mbp)	300.11
Average Len. (bp)	674.02
GC percent	39.44

integrity:start, indicates the number and percentage of genes that only contain start codons; integrity:end, indicates the number and percentage of genes that only contain stop codons; integrity:none, indicates the number and percentage of genes with no start codon and no stop codon; integrity:all, indicates the percentage of the number of complete genes (both start codons and stop codons); Total Len. (Mbp), indicates the total length of genes in the gene catalogue, in millions; Average Len. (bp), indicates the average length of genes in the gene catalogue; GC percent, indicates the predicted overall GC content of genes in the gene catalogue.

## Data Availability

All data generated or analysed during this study were included in this published article. The raw sequence data reported in this paper were deposited in the Genome Sequence Archive (Genomics, Proteomics and Bioinformatics 2021) in the National Genomics Data Center (Nucleic Acids Res 2022), China National Center for Bioinformation/Beijing Institute of Genomics, Chinese Academy of Sciences (GSA: CRA008466, CRA008470), and are publicly accessible at https://ngdc.cncb.ac.cn/gsa.

## References

[1] Rustam A. (2017). History of antimicrobial drug discovery: Major classes and health impact. Biochem. Pharmacol..

[2] Sandelin A., Hälli O., Härtel H., Herva T., Kaartinen L., Tuunainen E., Rautala H., Soveri T., Simojoki H. (2022). Effect of Farm Management Practices on Morbidity and Antibiotic Usage on Calf Rearing Farms. Antibiotics.

[3] Gnanadhas D.P., Marathe S.A., Chakravortty D. (2013). Biocides-resistance, cross-resistance mechanisms and assessment. Expert Opin. Investig. Drugs.

[4] Ouellette M., Kundig C. (1997). Microbial multidrug resistance. Int. J. Antimicrob. Agents.

[5] Alcalde-Rico M., Hernando-Amado S., Blanco P., Martínez J.L. (2016). Multidrug Efflux Pumps at the Crossroad between Antibiotic Resistance and Bacterial Virulence. Front. Microbiol..

[6] Besharati S., Motamedi H., Zallaghi R. (2018). A survey on microbial quality and antibiotic resistance in Karoon River, Khuzestan, Iran. Appl. Water Sci..

[7] Chiș A.A., Rus L.L., Morgovan C., Arseniu A.M., Frum A., Vonica-Țincu A.L., Gligor F.G., Mureșan M.L., Dobrea C.M. (2022). Microbial Resistance to Antibiotics and Effective Antibiotherapy. Biomedicines.

[8] Cloeckaert A., Schwarz S. (2001). Molecular characterization, spread and evolution of multidrug resistance in Salmonella enterica Typhimurium DT104. Vet. Res..

[9] Erol E., Scortti M., Fortner J., Patel M., Vázquez-Boland J.A. (2021). Antimicrobial Resistance Spectrum Conferred by pRErm46 of Emerging Macrolide (Multidrug)-Resistant *Rhodococcus equi*. J. Clin. Microbiol..

[10] Getahun Y.A., Ali D.A., Taye B.W., Alemayehu Y.A. (2022). Multidrug-Resistant Microbial Therapy Using Antimicrobial Peptides and the CRISPR/Cas9 System. Veter.-Med. Res. Rep..

[11] Khare T., Anand U., Dey A., Assaraf Y.G., Chen Z.S., Liu Z., Kumar V. (2021). Exploring Phytochemicals for Combating Antibiotic Resistance in Microbial Pathogens. Front. Pharmacol..

[12] Liu W., Liu Y.T. (2021). Roles of Multidrug Resistance Protein 4 in Microbial Infections and Inflammatory Diseases. Microb. Drug Resist..

[13] Lo C.C., Liao W.Y., Chou M.C., Wu Y.Y., Yeh T.H., Lo H.R. (2022). Overexpression of Resistance-Nodulation-Division Efflux Pump Genes Contributes to Multidrug Resistance in *Aeromonas hydrophila* Clinical Isolates. Microb. Drug Resist..

[14] Souza K.L.D., Fuzatti J.V.S., Camargo R.C., Pinto M.D., Silva T.K., Frias D.F.R. (2020). Prevalence of multidrug-resistant bacteria in the nasal cavity of horses asymptomatic for respiratory diseases. Rev. Univap..

[15] Van Veen H.W., Konings W.N. (1998). The ABC family of multidrug transporters in microorganisms. Biochim. Biophys. Acta (BBA)—Bioenerg..

[16] Yewale P.P., Lokhande K.B., Sridhar A., Vaishnav M., Khan F.A., Mandal A., Swamy K.V., Jass J., Nawani N. (2020). Molecular profiling of multidrug-resistant river water isolates: Insights into resistance mechanism and potential inhibitors. Environ. Sci. Pollut. Res..

[17] Sun K., Zhang J.M., Jiang D.W., Wang X., Ge Y., Deng X. (2020). Progress and Countermeasures of Antimicrobial Resistance of Animal Origin Bacterial Pathogens in China. J. Agric. Sci. Technol..

[18] Chen C., Zhou Y., Fu H., Xiong X., Fang S., Jiang H., Wu J., Yang H., Gao J., Huang L. (2021). Expanded catalog of microbial genes and metagenome-assembled genomes from the pig gut microbiome. Nat. Commun..

[19] Zhou Y.Y. (2021). Construction and Application of the Catalogs of Microbial Genomes, Genes and Antimicrobial Resistance Genes from Pig Gut Microbiome.

[20] Lu Z., Shen Q.C., Zhang C.P., Qi Z. (2021). Predictive analysis of whole genome sequencing for Salmonella serotype and antimicrobial resistance phenotypes. Acta Microbiol. Sin..

[21] Chen K., Pachter L. (2005). Bioinformatics for whole-genome shotgun sequencing of microbial communities. PLoS Comput. Biol..

[22] Handelsman J., Rondon M.R., Brady S.F., Clardy J., Goodman R.M. (1998). Molecular biological access to the chemistry of unknown soil microbes: A new frontier for natural products. Chem. Biol..

[23] Tringe S.G., Rubin E.M. (2005). Metagenomics: DNA sequencing of environmental samples. Nat. Rev. Genet..

[24] Tringe S.G., von Mering C., Kobayashi A., Salamov A.A., Chen K., Chang H.W., Podar M., Short J.M., Mathur E.J., Detter J.C. (2005). Comparative metagenomics of microbial communities. Science.

[25] Raes J., Foerstner K.U., Bork P. (2007). Get the most out of your metagenome: Computational analysis of environmental sequence data. Curr. Opin. Microbiol..

[26] Liu P., Jiang Y., Gu S., Xue Y., Yang H., Li Y., Wang Y., Yan C., Jia P., Lin X. (2021). Metagenome-wide association study of gut microbiome revealed potential microbial marker set for diagnosis of pediatric myasthenia gravis. BMC Med..

[27] Li J., Hao H., Cheng G., Liu C., Ahmed S., Shabbir M.A., Hussain H.I., Dai M., Yuan Z. (2017). Microbial shifts in the intestinal microbiota of Salmonella infected chickens in response to enrofloxacin. Front. Microbiol..

[28] De Smet J., Boyen F., Croubels S., Rasschaert G., Haesebrouck F., Temmerman R., Rutjens S., De Backer P., Devreese M. (2020). The impact of therapeutic-dose induced intestinal enrofloxacin concentrations in healthy pigs on fecal *Escherichia coli* populations. BMC Vet. Res..

[29] Xiao L., Estellé J., Kiilerich P., Ramayo-Caldas Y., Xia Z., Feng Q., Liang S., Pedersen A.Ø., Kjeldsen N.J., Liu C. (2016). A reference gene catalogue of the pig gut microbiome. Nat. Microbiol..

[30] Wang C., Li P., Yan Q., Chen L., Li T., Zhang W., Li H., Chen C., Han X., Zhang S. (2019). Characterization of the Pig Gut Microbiome and Antibiotic Resistome in Industrialized Feedlots in China. mSystems.

[31] Van Boeckel T.P., Pires J., Silvester R., Zhao C., Song J., Criscuolo N.G., Gilbert M., Bonhoeffer S., Laxminarayan R. (2019). Global trends in antimicrobial resistance in animals in low- and middle-income countries. Science.

[32] McArthur A.G., Waglechner N., Nizam F., Yan A., Azad M.A., Baylay A.J., Bhullar K., Canova M.J., De Pascale G., Ejim L. (2013). The Comprehensive Antibiotic Resistance Database. Antimicrob. Agents Chemother..

[33] Chen T., Chen X., Zhang S., Zhu J., Tang B., Wang A., Dong L., Zhang Z., Yu C., Sun Y. (2021). The Genome Sequence Archive Family: Toward Explosive Data Growth and Diverse Data Types. Genom. Proteom. Bioinform..

[34] Xue Y., Bao Y., Zhang Z., Zhao W., Xiao J., He S., Zhang G., Li Y., Zhao G., Chen R. (2022). Database Resources of the National Genomics Data Center, China National Center for Bioinformation in 2022. Nucleic Acids Res..

[35] Wen Y., Li S., Wang Z., Feng H., Yao X., Liu M., Chang J., Ding X., Zhao H., Ma W. (2022). Intestinal Microbial Diversity of Free-Range and Captive Yak in Qinghai Province. Microorganisms.

